# Pathophysiology of Hyperechogenic Bowel in Congenitally Human Cytomegalovirus Infected Fetuses

**DOI:** 10.3390/microorganisms8050779

**Published:** 2020-05-22

**Authors:** Liliana Gabrielli, Maria P. Bonasoni, Angela Chiereghin, Giulia Piccirilli, Eva C. Borgatti, Giuliana Simonazzi, Nunzio C. M. Salfi, Ione Tamagnini, Tiziana Lazzarotto

**Affiliations:** 1 Operative Unit of Clinical Microbiology, St. Orsola Polyclinic, University of Bologna, Via Massarenti 9, 40138 Bologna, Italy; giulia.piccirilli2@unibo.it; 2 Pathology Unit, Arcispedale Santa Maria Nuova, Azienda USL-IRCCS, Viale Risorgimento 80, 42123 Reggio Emilia, Italy; MariaPaola.Bonasoni@ausl.re.it (M.P.B.); Ione.Tamagnini@ausl.re.it (I.T.); 3 Department of Specialized, Experimental, and Diagnostic Medicine, Operative Unit of Clinical Microbiology, St. Orsola Polyclinic, University of Bologna, Via Massarenti 9, 40138 Bologna, Italy; angela.chiereghin2@unibo.it (A.C.); caterina.borgatti92@gmail.com (E.C.B.); tiziana.lazzarotto@unibo.it (T.L.); 4 Department of Obstetrics and Gynecology, St. Orsola Polyclinic, University of Bologna, Via Massarenti 9, 40138 Bologna, Italy; giuliana.simonazzi@unibo.it; 5 Pathology Unit, St. Orsola Polyclinic, University of Bologna, Via Massarenti 9, 40138 Bologna, Italy; nunziosalfi@gaslini.org

**Keywords:** CMV, hyperechogenic bowel, ganglion cells, bcl-2, meconium

## Abstract

Hyperechogenic bowel (HB) is a nonspecific ultrasound finding that can be associated with human cytomegalovirus (CMV) congenital infection. In this study, we investigated HB pathophysiology in CMV-infected fetuses. We examined small and large intestine as well as pancreas in 8 fetuses at 22 weeks of gestation with congenital CMV infection. Ultrasound findings showed 4 fetuses with HB and 4 without. As negative group, 4 fetuses without CMV infection and without HB were studied. Immunohistochemistry for CMV, lymphocytic infiltrate, B-cell leukemia/lymphoma-2 (bcl-2), CD-117, cystic fibrosis transmembrane regulator (CFTR) were performed. HB fetuses showed multiple and sequential CMV-positive ganglion cells of Auerbach’s myenteric plexus. In the ganglia, bcl-2 was weakly expressed representing a reduced neuronal functionality. CD-117 revealed a regular distribution of Cajal cells, the pacemakers of intestinal contractility. Pancreas showed normal CFTR staining, indicating a preserved exocrine secretion, thus unlikely a contributory factor in HB. In CMV-infected fetuses without HB, CMV-positive cells were scatteredly found in ganglion cells and bcl-2 was strongly expressed. Intestinal CD-117 and pancreatic CFTR expression were similar to fetuses with HB. In conclusion, fetal CMV infection of the bowel may lead to peristalsis impairment (paralytic ileus) due to intestinal plexus involvement, which at ultrasound appeared as HB.

## 1. Introduction

Human cytomegalovirus (CMV) is the most common congenital infection affecting 0.5–2% of all live births and is a leading cause of hearing and central nervous system impairments in children [1,2]. Maternal primary infection seems to represent a high risk of transmission and severity of fetal infection, but it has now been recognized that congenital infection and fetal disability also follow non-primary maternal infection [2,3]. Detection of CMV DNA in the amniotic fluid is the gold standard for prenatal diagnosis. Amniocentesis should be performed after 20–21 weeks of gestation and at least 8 weeks after estimated maternal seroconversion. When the diagnosis of fetal infection is confirmed through amniocentesis, the prognostic evaluation of fetal infection relies on imaging, using a combination of ultrasound and cerebral magnetic resonance imaging [4]. Prenatal ultrasound findings may be cerebral, such as ventriculomegaly, microcephaly and periventricular leukomalacia, as well as non-cerebral, such as hyperechogenic bowel (HB), enlargement of liver and spleen, ascites, hydrops, pericardial effusion, and placental enlargement [5]. The extracerebral manifestations are unspecific and show that the entire fetal body can be affected as a result of the affinity of the virus to endothelial and epithelial cells [6]. Leruez-Ville M et al. reviewed ultrasound findings in fetal infection with CMV in the literature and observed that the most frequent ultrasound anomaly was HB (82 out of 637 cases; 13%) [2].

Fetal HB is a soft marker found on second trimester sonography with an incidence of 0.2–1.8% [7]. It is defined as bowel of similar or greater echogenicity than surrounding bone. This sonographic finding is nonspecific and the vast majority of fetuses are normal, however it also has been associated with several pathologic conditions that include cystic fibrosis, intraamniotic bleeding, chromosomal abnormalities and in utero infection such as CMV, parvovirus B19 and *Toxoplasma gondii* infection [8,9]. In a systematic review [7], congenital infections occurred in 2.2% of fetuses with HB and CMV was the most common congenital infection occurring in 1.4% of cases, while the incidence of parvovirus B19 and *Toxoplasma gondii* infections was 0.9% and 0.6%, respectively.

The pathophysiology of HB is likely heterogeneous. In most cases the increased echogenicity is thought to be due to abnormal, highly viscous meconium within the small bowel caused by abnormal pancreatic enzymatic secretion (i.e., cystic fibrosis) or poor bowel motility resulting in increased water absorption (i.e., trisomy 21) [10,11,12]. Focal areas of bowel echogenicity have also been associated with intestinal ischemia. Moreover, HB may be due to blood swallowing as blood is extremely echogenic [9,10,12].

The pathophysiology of the association between HB and fetal infections is still unknown. Regarding parvovirus B19 infection, Jouannic et al. suggest a direct viral induced damage of the fetal bowel through a possible cytotoxic effect on endothelial cells leading to ischemia. In addition, this direct effect of parvovirus B19 on the fetal intestinal wall may be associated with inflammatory response with edema and histological remodelling. Along with the severity of parvovirus B19 induced intestinal injury, the fetus may present transient HB with spontaneous resolution or severe bowel ischemia with perforation leading to meconial peritonitis [13]. Regarding CMV infection, in only one paper the physiopathologic mechanism underlying HB was studied histologically. Dechelotte reported three cases of pseudo-meconium ileus due to CMV infection of ganglion cells suggesting a paralytic ileus [14]. Other authors only speculated that CMV might directly damage the enteric mucosa as a consequence of physiologic amniotic fluid swallowing containing CMV [15].

In this study, we aimed to investigate thoroughly the pathophysiology of increased bowel echogenicity in CMV-infected fetuses. We compared CMV-infected fetuses with and without HB identified by ultrasound at 20–21 weeks gestation. We evaluated histological features and we performed immunohistochemistry for CMV, inflammatory infiltrate, markers of neuronal vitality, intestinal pacemaker activation and pancreatic enzymatic secretion in order to study intestinal motility and pancreatic functionality.

## 2. Materials and Methods

### 2.1. Study Population

Eight fetuses with congenital CMV infection documented at 20–21 weeks gestation by invasive positive prenatal diagnosis, underwent histological examination after elective termination of pregnancy. In particular, we focused on the examination of small and large intestines, as well as the pancreas. All fetuses were from pregnant women with primary CMV infection arising before the twelfth week of gestation. Women who had anti-CMV IgM and anti-CMV IgG of low avidity or who seroconverted to CMV IgG positivity were classified as having primary infection [16,17,18]. The diagnosis of fetal CMV infection was based on CMV positivity in amniotic fluid by real-time PCR at 20–21 weeks of gestation. The viral load was >10^5^ copies/mL in all amniotic fluids. At the time of amniocentesis, all pregnant women underwent ultrasound examinations that included a survey of all fetal organs [19]. According to ultrasound findings, CMV-infected fetuses were classified in two groups: fetuses with HB and fetuses without HB (Table 1).

As negative group, we studied four fetuses of the same gestational period without HB and without CMV infection. The fetuses were from spontaneous miscarriages, in particular, two were from cervical incompetence and two from placental abruption with no congenital malformations or chromosomal abnormalities. All women were CMV-seronegative at the time of delivery.

The fetal tissues were analyzed after informed consent had been obtained from the parents and according to the policies of the Ethical Committee of St. Orsola Polyclinic, Bologna, Italy (approval numbers: 14/2017/U/tess and 8/2010/O/Sper) and regulations of the Italian Ministry of Health, Rome, Italy. The study was also conducted according to the principles of the Declaration of Helsinki.

### 2.2. Histological Examination

Fetal tissues were fixed in buffered 4% formaldehyde, and haematoxylin and eosin standard sections were obtained from paraffin-embedded blocks of fetal organs. In particular, serial sections of jejunum, ileum, large bowel and pancreas were histologically examined.

Immunohistochemistry for CMV immediate early, early and late antigens was performed to identify CMV-positive cells. Inflammatory infiltrate was evaluated with antibodies for CD3 (T Lymphocytes), CD4 (T-helper lymphocytes), CD8 (T-cytotoxic lymphocytes), and CD20 (B lymphocytes) [20,21]. B-cell leukemia/lymphoma-2 (bcl-2) and CD-117 (c-KIT) expression was assessed for intestinal ganglion cells vitality and Cajal cells localization, respectively. Cystic fibrosis transmembrane regulator (CFTR) expression was studied to assess membrane chloride channels functionality in pancreas.

The following antibodies were used: anti-CMV (clones 8B1.2, 1G5.2, 2D4.2) mouse monoclonal primary antibody (Cell Marque, Rocklin, USA); anti-CD3 (clone 2GV6), anti-CD4 (clone SP35), anti-CD8 (clone SP57), anti-c-KIT (clone 9.7) rabbit monoclonal primary antibodies (Ventana Group, Milano, Italy); anti-CD20 (clone L26), anti-bcl-2 (clone 124) mouse monoclonal primary antibodies (Ventana Group, Milano, Italy); anti-CFTR (clone CF3) mouse monoclonal primary antibody (Histo-Line Laboratories, Milano, Italy).

Formalin-fixed, paraffin-embedded tissue sections were obtained and processed in a Benchmark Ultra Immunostainer (Ventana Medical Systems, Tucson, AZ, USA) and visualization of the immunological reaction was obtained with OptiView DAB Detection kit (Ventana Medical Systems, Tucson, AZ, USA); slides were counterstained with haematoxylin and bluing reagents.

### 2.3. Virological Examination

DNA was extracted from amniotic fluid (1 mL eluted in 25 μL of elution buffer) with the NucliSens easyMAG System (bioMerieux, Marcy l’Etoile, France). CMV DNA was quantified with a real-time PCR assay (CMV ELITe MGB kit, ELITechGroup, Turin, Italy), according to the manufacturer’s package insert. Amplification, detection and analysis were performed with the ABI PRISM 7500 platform (Applied Biosystems). The detection limit was 11 copies/reaction and viral load was reported as number of copies/mL [22].

## 3. Results

We studied eight fetuses at 21–22 weeks of gestation from elective termination of pregnancy. All fetuses had congenital CMV infection documented by invasive prenatal diagnosis disclosing high viral load in the amniotic fluid. According to prenatal ultrasound findings, the fetuses were divided in the following two groups: CMV-infected fetuses with HB and CMV-infected fetuses without HB. A third group designated as negative group, constituted by fetuses of the same gestational period without HB and without CMV infection, was investigated. Autopsy findings in the 3 groups studied are summarized in Table 2.

### 3.1. CMV-Infected Fetuses with Prenatal Hyperechogenic Bowel at Ultrasound

In this group, three out of four fetuses presented prenatal ultrasound brain abnormalities other than HB. In particular, one fetus had microcephaly, one periventricular hyperechogenicity and one ventriculomegaly.

**Figure 1:**
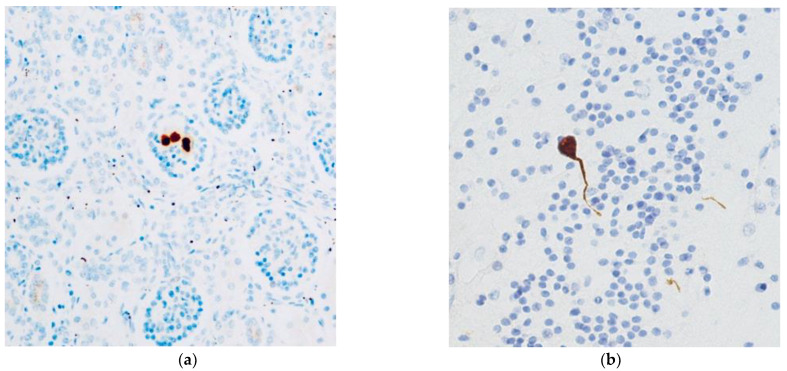
CMV-positive cells in kidney (**a**) and brain (**b**) (CMV-IHC; 4HPF) related to a widespread infection.

At histology, all fetuses had a multiorgan CMV infection confirmed by immunohistochemistry. In particular, the following fetal organs were CMV-positive: lung, kidney, liver, pancreas and brain (Figure 1). Two out of four fetuses had CMV-positive heart.

**Figure 2:**
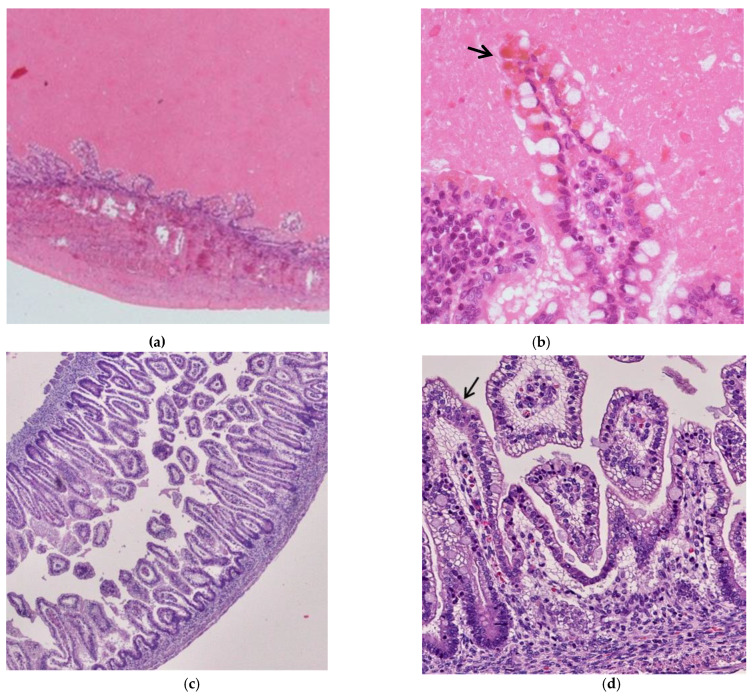
Intestinal wall comparison between CMV-infected fetuses with and without HB. CMV-infected fetuses with HB: (**a**) The intestinal villi were compressed and flattened by intraluminal meconium (HE staining; 2HPF); (**b**) The enterocytes showed brown pigments (arrow) in the cytoplasm due to meconium absorption (HE staining; 10HPF). CMV-infected fetuses without HB: (**c**) Intestinal wall without meconium in the lumen showing elongated and finger-like villi (HE staining; 2HPF); (**d**) The enterocytes displayed clear cytoplasm (HE staining; 10HPF).

Regarding the intestine, two fetuses investigated macroscopically showed dilatation of the distal intestine and meconium was thickened, dark green and adherent to the intestinal mucosa. Microscopically, the small intestine was markedly dilated, the lumen contained inspissated meconium which compressed the intestinal wall with flattened villi (Figure 2a). The enterocytes showed brown pigments in the cytoplasm, compatible with meconium absorption (Figure 2b). As comparison, a normal intestinal wall was shown in Figure 2c,d.

In the other two fetuses, no grossly intestinal dilatation was found, but the meconium was thickened and adherent to the intestinal mucosa wall. However, histologically villi were compressed with brown pigments in the cytoplasm.

**Figure 3:**
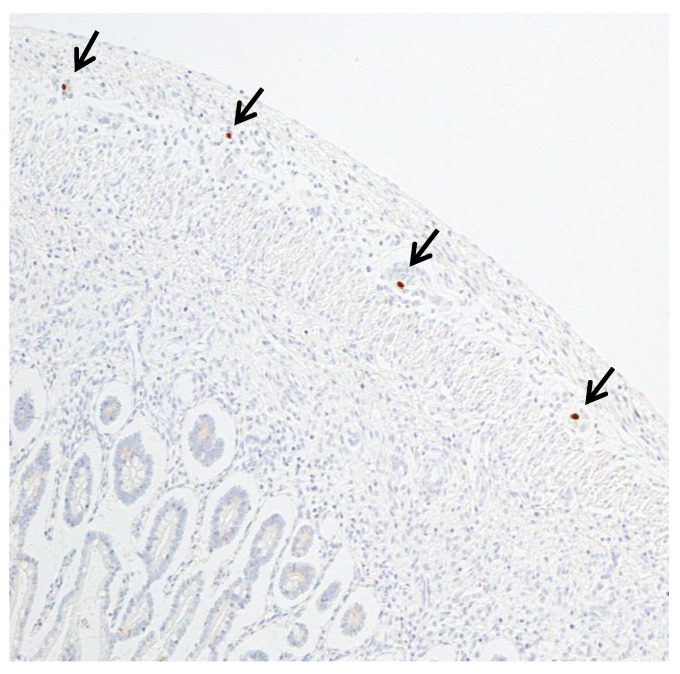
Multiple ganglion cells in Auerbach’s myenteric plexus were CMV-positive (arrows) (CMV-IHC; 2HPF).

In these four fetuses, immunohistochemistry revealed multiple and sequential ganglion cells of Auerbach’s myenteric plexus that were CMV-positive (Figure 3), mainly surrounded by CD-4 and CD-8 T lymphocytes equally represented (data not shown).

Scattered CMV-positive ganglion cells in Meissner’s submucosal plexus and in endothelial cells were observed. However, no evidence of necrosis and apoptosis were found in the intestinal wall.

**Figure 4:**
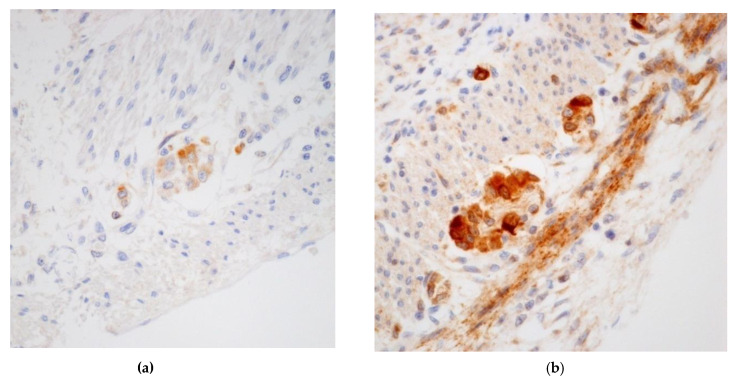
(**a**) In CMV-infected fetuses with HB, bcl-2 was weakly expressed and not in all ganglion cells (bcl-2-IHC; 10HPF); (**b**) In CMV-infected fetuses without HB, bcl-2 was strongly expressed in ganglion cells (bcl-2-IHC; 10HPF).

In the ganglia, bcl-2 was weakly expressed (Figure 4a). CD-117 staining showed a normal population of Cajal cells.

Regarding the pancreas, CMV-positive cells were mainly epithelial surrounded by CD-8 T lymphocytes with rare CD-4T lymphocytes (data not shown). CFTR staining was normally expressed in the epithelial cells.

### 3.2. CMV-Infected Fetuses with No Prenatal Hyperechogenic Bowel at Ultrasound

In this group, one out of 4 fetuses presented at prenatal ultrasound cerebral periventricular hyperechogenicity. Even in this group, all fetuses had a multiorgan CMV infection confirmed by immunohistochemistry. In particular, the following fetal organs were CMV-positive: lung, kidney, liver, pancreas and brain. Heart was CMV-positive in two out of the four fetuses.

Regarding the intestine, all fetuses had no dilatation of the distal intestine and no meconium stasis in the intestinal lumen. Microscopically, the intestinal villi were well elongated appearing as finger-like projections extended into the lumen. CMV-positive cells were found in endothelial cells and in 2 cases also in scattered ganglion cells of both plexuses. Scattered CD-4 and CD-8 T lymphocytes equally represented were observed (data not shown). In these 4 fetuses, bcl-2 was strongly expressed in ganglion cells (Figure 4b) and CD-117 showed a regular distribution.

In the pancreas, CMV-positive cells were mainly epithelial and CFTR was normal expressed similarly to fetuses with HB. Inflammatory infiltrate was mainly composed of CD-8 T lymphocytes and few CD-4 T lymphocytes (data not shown).

### 3.3. Negative Group

In these four fetuses, no dilatation of distal intestine was observed. No CMV-positive cells were detected in intestine and pancreas as well as in the other fetal organs. The intestinal villi appeared normal extending as finger-like projections into the lumen with no evidence of meconium stasis.

In the intestine, lymphocytic infiltrate of CD-8 and rare CD-4 T lymphocytes was physiologically present in the mucosa. Bcl-2 was strongly expressed in ganglion cells and CD-117 showed a regular distribution. In the pancreas, CFTR was regularly expressed and rare CD-8 and CD-4 T lymphocytes were observed.

### 3.4. Summary

CMV-infected fetuses with no prenatal HB presented the same intestinal and pancreatic features of the negative group, except for the presence of scattered CMV-positive cells. Only in CMV-infected fetuses with prenatal HB, villi were flattened with meconium granules in the cytoplasm, sequential ganglion cells of Auerbach’s myenteric plexus were CMV-positive, and in the ganglia bcl-2 was weakly expressed indicating a reduced neuronal functionality. In all fetuses, pancreatic exocrine secretion was preserved as shown by CFTR staining.

## 4. Discussion

In this study we investigated the pathophysiology of CMV-related HB evaluating intestinal motility and pancreatic functionality in congenitally CMV-infected fetuses.

In fetal CMV infection, abnormal ultrasound findings can be differentiated between cerebral and extracerebral signs [2,23]. Increased echogenicity of fetal bowel is a typical extracerebral manifestation observed in 13% of infected fetuses [2]. This sonographic finding is considered nonspecific and the vast majority of fetuses are normal [7], however, when associated with CMV infection, this sign is consistent with extensive spreading of CMV within the fetus [15]. Extracerebral ultrasound findings after maternal CMV infection occurred before 14 weeks of gestation are associated with a high risk of developing sensorineural hearing loss and brain lesions [2].

The development of HB has different causes. HB in aneuploidy (i.e., trisomy 21) is thought to be due to decreased bowel motility with increased water absorption from the meconium [12]. In fetuses with isolated HB, chromosomal anomalies occur in 3.3% of cases [7]. In fetuses with cystic fibrosis, HB may be due to modifications in the consistency of meconium in the small intestine as a result of abnormalities in pancreatic enzyme secretion [12]. Among fetuses with isolated HB, 2.2% present cystic fibrosis [7].

It is still uncertain how a viral infection results in the echogenic appearance of the bowel. It may be caused by inflammation, meconium peritonitis, ascites or anemia [12]. The association of congenital infections with isolated HB has been reported in 2.2% and the most commonly detected infectious agent is CMV [7,24,25].

As far as we know, the only histological study which examines fetal CMV infection and HB was carried out by Dechelotte et al. in 1992 [14]. They analyzed three cases of prenatal ileus associated with CMV infection. In all cases CMV was found in ganglion cells of myenteric and submucosal plexuses along the small and large intestine. The authors suggested that CMV caused paralytic ileus, but they did not evaluate in depth neuronal vitality and pancreatic secretion functionality.

We briefly reviewed the anatomical organization of the intestinal wall to better understand better our results and explain potential CMV involvement in the pathogenesis of HB.

**Figure 5:**
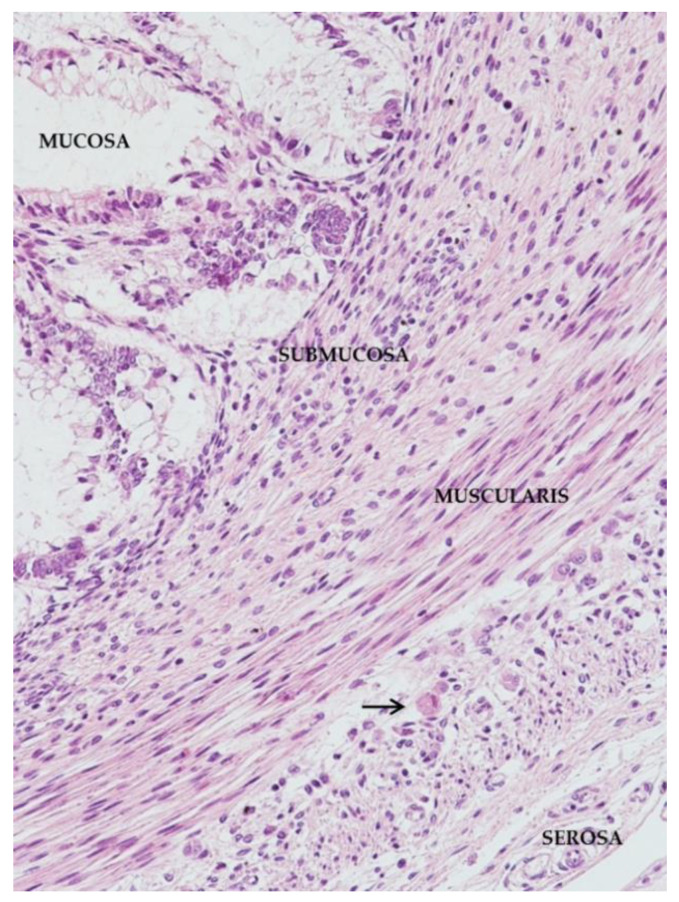
Whole section of the wall of large bowel from the mucosa (**top left**) to the serosa (**bottom right**). A cytomegalic cell is present (arrow) within the Auerbach’s plexus (HE staining; 4HPF).

The intestinal wall is divided in the following layers: mucosa, submucosa, muscularis propria, and serosa (Figure 5).

The intestinal mucosa provides the maximal surface area for the purpose of nutrient absorption. Histologically, the mucosa is composed of numerous villi, which extend into the intestinal lumen as finger-like projections, covered by epithelial cells (enterocytes). The submucosa contains the submucosal neural plexus (Meissner plexus) that controls local secretion, absorption, and muscle movements. The muscularis propria is composed of a thick layer of smooth muscle organized in an inner circular and an outer longitudinal coat. Between the two coats reside the Auerbach myenteric plexus. This plexus increases the tone of the gut and the velocity and intensity of contractions, being more involved in peristalsis control. Meissner plexus and Auerbach myenteric plexus constitute the enteric nervous system that contains 200–600 million neurons, distributed in many thousands of small ganglia. In addition to neural plexus, the interstitial cells of Cajal are critical for bowel motility. These cells express CD-117, a tyrosine kinase receptor [26].

In our study we examined eight fetuses at 21–22 weeks of gestation with congenital CMV infection and we focused our investigation on the intestine and pancreas in order to shed light over the pathogenesis of CMV-related bowel echogenicity.

In 4 CMV-infected fetuses, HB was not detected by prenatal ultrasound. All fetuses had no dilatation of the distal intestine and no meconium stasis in the intestinal lumen. Microscopically, the intestinal villi were well elongated appearing as finger-like projections extended into the lumen. CMV-positive cells were found in endothelial cells and in 2 cases also in scattered ganglion cells of Meissner plexus and Auerbach’s myenteric plexus.

In the other 4 CMV-infected fetuses prenatal ultrasound performed at 21 weeks of gestation disclosed HB. Macroscopically thickened meconium was found in all of them, associated with bowel dilatation in two fetuses. Microscopically, meconium stasis was evident with flattened villi and meconium granules in the cytoplasm of enterocytes. With immunohistochemistry, scattered CMV-positive ganglion cells in Meissner’s submucosal plexus and in endothelial cells were observed. In addition, multiple and sequential CMV-infected ganglion cells of Auerbach’s myenteric plexus were identified.

Meconium consists of desquamated epithelial cells from the intestine and skin, bile, pancreatic secretions, and the residue of swallowed amniotic fluid. Accumulation begins as early as the twelfth gestational week, when fetal swallowing commences. This condition is physiological during fetal life. The meconium sometimes becomes thickened and congested in the intestine, a condition known as meconium ileus. Usually it is correlated with cystic fibrosis in which inspissated meconium is due to a deficiency in pancreatic enzymes and abnormal mucin production [27]. Moreover, thickened meconium can be caused by poor bowel motility [12].

In our four fetuses with HB, we observed the presence of thickened meconium and meconium stasis. The presence of sequential CMV-infected ganglion cells of Auerbach’s myenteric plexus suggests peristalsis impairment. Similarly, Dechelotte described 3 cases of prenatal meconium ileus associated with congenital CMV infection. Cytomegalic cells were found in endothelial cells and in the myenteric and submucosal plexuses, suggesting that the ileus was probably paralytic and due to intestinal plexuses involvement [14]. Our results were similar, assuming paralytic ileus due to CMV as the cause of HB.

In addition, in our study, we evaluated CFTR staining to exclude abnormal pancreatic enzymatic secretion in the pathogenesis of thickened meconium. CFTR is an anion selective transmembrane ion channel that mainly regulates chloride transport. Cystic fibrosis is caused by mutations of the CFTR gene and consequently to the malfunction of the CFTR protein in the epithelial cells of the pancreas resulting in enzyme insufficiency and thickening of gastrointestinal secretions [28]. In all the fetuses involved in our groups, in the pancreas, CFTR staining was normal, indicating that chloride channels were preserved and the exocrine function was maintained. These results exclude pancreatic exocrine insufficiency as the cause of HB. Therefore, HB is probably caused by ganglion cells infection leading to peristalsis impairment.

Moreover, reduced functionality of ganglion cells in HB fetuses was confirmed by immunohistochemistry of bcl-2. Expression of bcl-2 in enteric ganglion cells of the myenteric and submucous plexuses is displayed in the fetus and during childhood and is also retained in adult bowel. The bcl-2 protein is localized in the inner mitochondria membranes and has been found to have the specific functional role of blocking programmed cell death, or apoptosis, suggesting a role in neuronal survival [29]. We observed that bcl-2 was strongly expressed in negative group and in CMV-infected fetuses with no HB, while weakly expressed and not in all ganglion cells in fetuses with HB. This may suggest that infected ganglion cells could have reduced functionality. Similarly, in chronic intestinal pseudo-obstruction syndrome, characterized by impaired gastrointestinal propulsion, a reduced bcl-2 expression was described [30].

We also investigated Cajal cells, which play a key role in bowel motility. There are various types with different functions. Myenteric interstitial cells of Cajal serve as a pacemaker, which creates the bioelectrical slow wave potential that leads to contraction of the smooth muscle. Intramuscular interstitial cells of Cajal are involved in the stimulation of smooth muscle cells [31,32,33]. In all groups of fetuses, Cajal cells were regularly represented. Therefore, HB is not related to pacemaker altered function or abnormal induction of smooth muscle cells contraction.

Regarding inflammatory infiltrate, we characterized the inflammatory infiltrate and we observed that CMV-positive cells were surrounded by CD-4 and CD-8 T lymphocytes, equally represented. Dechelotte et al. observed a chronic ganglionitis in the plexuses composed of lymphocytes, but it was not further characterized [14]. Similarly, to our study, enteric inflammatory neuropathies are characterized by an inflammatory infiltrate composed of both CD4 and CD8 lymphocytes mainly confined to the myenteric plexus [34].

In our study, although few cases were examined, we demonstrated for the first time that in CMV-infected fetuses with HB, the ileus is probably paralytic as a result of intestinal plexus involvement only. In fact, we found multiple and sequential CMV-infected ganglion cells of Auerbach’s myenteric plexus with a reduced bcl-2 expression indicating impaired functionality. On the other hand, in the pancreas CFTR staining was normal, indicating that the exocrine function was maintained, thus unlikely a contributory factor in HB. Overall, fetal CMV infection of the bowel may lead to peristalsis impairment (paralytic ileus) that at ultrasound appeared as HB.

## 5. Conclusions

HB is a nonspecific ultrasound finding in second trimester, but when associated with fetal CMV infection, it is correlated with widespread multiorgan dissemination of the virus. Infection of multiple ganglion cells may cause an impairment in intestinal functionality, and overall a reduced intestinal motility with meconium stasis. The ileus should be considered transient, in fact, congenitally CMV-infected newborns do not present intestinal involvement [35]. This is probably explained by the absence of neuronal necrosis, which allows intestinal motility to recover.

## Figures and Tables

**Table 1:** Diagnosis of maternal and fetal CMV infection in the study population.

Case	Age (Years Old)	Diagnosis of Primary CMV Infection	Viral Load in AF at 21 WG (Copies/mL)	Cerebral US Abnormalities at 21 WG	Extracerebral US Abnormalities at 21 WG
1	33	CMV IgG seroconversion	>5.000.000	microcephaly	HB
2	20	CMV IgM + CMV IgG + low avidity CMV IgG	1.700.000	periventricular hyperechogenicity	HB
3	29	CMV IgM + CMV IgG + low avidity CMV IgG	>5.000.000	ventriculomegaly	HB
4	37	CMV IgM + CMV IgG + low avidity CMV IgG	988.460	-	HB
5	34	CMV IgM + CMV IgG + low avidity CMV IgG	790.000	periventricular hyperechogenicity	-
6	31	CMV IgG seroconversion	>5.000.000	-	-
7	32	CMV IgG seroconversion	>5.000.000	-	-
8	22	CMV IgM + CMV IgG + low avidity CMV IgG	1.250.000	-	-

CMV: human cytomegalovirus; AF: amniotic fluid; WG: weeks of gestation; US: ultrasound; -: normal US findings.

**Table 2:** Autopsy findings in the 3 groups studied.

Fetuses	Case	Macroscopic Findings	Bowel IHC CMV	Bowel IHC bcl-2	Bowel IHC CD-117	Pancreas IHC CFTR
**CMV-infected** **with** **USHB**	1	intestinal dilatation thickened meconium	ganglion cells (multiple in AP, scattered in MP) endothelial cells (scattered)	-/+	+	+
	2	thickened meconium	ganglion cells (multiple in AP, scattered in MP) endothelial cells (scattered)	-/+	+	+
	3	intestinal dilatation thickened meconium	ganglion cells (multiple in AP, scattered in MP) endothelial cells (scattered)	-/+	+	+
	4	thickened meconium	ganglion cells (multiple in AP, scattered in MP) endothelial cells (scattered)	-/+	+	+
**CMV-infected** **without** **USHB**	5	normal	ganglion cells (scattered in AP and MP) endothelial cells (scattered)	+	+	+
	6	normal	ganglion cells (scattered in AP and MP) endothelial cells (scattered)	+	+	+
	7	normal	endothelial cells (scattered)	+	+	+
	8	normal	endothelial cells (scattered)	+	+	+
**Uninfected** **without** **USHB**	9	normal	-	+	+	+
	10	normal	-	+	+	+
	11	normal	-	+	+	+
	12	normal	-	+	+	+

CMV: human cytomegalovirus; US: ultrasound; HB: hyperechogenic bowel; AP: Auerbach’s myenteric plexus; MP: Meissner plexus; IHC: immunohistochemistry; -/+: weak expression at IHC; +: positivity, meaning regular expression at IHC; -: negative.
